# Sexual Abuse of Older Nursing Home Residents: A Literature Review

**DOI:** 10.1155/2015/902515

**Published:** 2015-01-06

**Authors:** Wenche Malmedal, Maria Helen Iversen, Astrid Kilvik

**Affiliations:** Faculty of Nursing, Sør-Trøndelag University College, Trondheim, Norway

## Abstract

Despite an increasing literature related to elder abuse, sexual abuse of older persons in general and of vulnerable adults living in nursing homes in particular is still sparsely described. The purpose of this study was to assess the state of knowledge on the subject of sexual abuse against older nursing home residents through a literature review. Systematic searches in reference databases including Cinahl, Medline, OVID Nursing Database, ISI Web of Science, PsycINFO, Cochrane Library, and SveMed + were conducted. Through several phases of selection of the articles, using strict inclusion and exclusion criteria, six articles were chosen for a deeper examination. Findings from the review show that sexual abuse occurs in nursing homes and that both older women and men are victims of sexual abuse. Perpetrators appear mainly to be staff and other residents and mainly to be men, but also women abuse both older men and older women. Findings from the literature review show that there is a need for knowledge and further research on the topic of sexual abuse against older residents in nursing homes. Furthermore, there is a need for good policies and reporting systems, as an important step in seriously addressing sexual abuse against older persons.

## 1. Introduction

The first studies of elder abuse were conducted in the 1970s [1, 2]. Since then, the research field has increased, and this increased interest in elder abuse has raised questions concerning definitions, methods, and theory [3]. At the present, there are no agreed universal or standardized definitions [4], and a review study from 2009 [5] demonstrates how differences in the definition and choice of measurement instrument can influence the prevalence rates found in a study. Recent studies [6–10] use five different categories of abuse: psychological, physical, sexual, financial, and neglect. This is according to the definition chosen by World Health Organization stated in The Toronto Declaration [11]. Abuse perpetrated against an older person is often not limited to only one form; for example, physical violence is often accompanied by psychological abuse, and financial abuse may be accompanied with neglect or physical abuse [12]. The WHO's World report on violence and health also states that “Regardless of the type of abuse, it will certainly result in unnecessary suffering, injury or pain, the loss or violation of human rights, and a decreased quality of life for the older person” [13, page 126].

Even though there is evidence that abuse and neglect are a common aspect of life in nursing homes in many countries [14–19], systematic reviews of the literature conclude that elder abuse research is minimal and that prevalence in nursing homes is difficult to estimate [3, 20].

WHO concludes that surveys have shown that between 4 and 6% of older people experience some form of abuse in their homes and that mistreatment in institutions may be more extensive than generally believed [13].

Sexual abuse defined as “nonconsenting sexual contact of any kind” is said to be the most hidden form of elder abuse [21] and is the least acknowledged and reported type of elder mistreatment. Sexual contact with any person incapable of giving consent is also considered sexual abuse. The definition of sexual abuse includes (but is not limited to) unwanted touching and all types of sexual assault or battery, such as rape, sodomy, coerced nudity, and sexually explicit photographing [22].

On global basis, there are no official prevalence statistics of sexual abuse of older persons. A prevalence survey report from UK shows that 0.3% had experienced one or more instances of sexual abuse since the age of 65 [8], and a prevalence study conducted in USA shows that 0.6% of older persons are exposed to sexual abuse [23]. A recently conducted prevalence study from a district in Sweden [6] reports that 2.2% of the woman and 1.2% of the men had been exposed to sexual abuse after the age of 65.

Nursing homes are not free from sexual abuse [24–26], and both staff and residents have been identified as perpetrators. Residents in nursing homes are particularly vulnerable and at risk of abuse because they are more or less dependent on their caregivers owing to chronic illnesses, especially those with cognitive and behavioral problems [27–29].

Attention was drawn to this type of abuse during the 1990s [30–32]. In 1999 a conference under the title “The Great Taboo” was conducted by Action on Elder Abuse. Despite an increasing literature related to elder abuse, sexual abuse of older persons in general and of vulnerable adults living in nursing homes in particular is still sparsely described.

The small percentage of sexual abuse reported in studies of elder abuse may be due to the fact that many categorize sexual abuse under physical abuse when they report acts of abuse. This has hindered systematic studies of sexual abuse [21].

The main purpose of this study was to answer the following: What knowledge do we have about sexual abuse of older nursing home residents? The concrete research questions were the following:

1. How is sexual abuse defined in the studies?
2. What knowledge do we have about prevalence and types of sexual abuse in nursing homes?
3. What knowledge do we have about the characteristics of the victims and perpetrators?
4. What do we know about the consequences for the victims and perpetrators?
5. Do we know anything about how the nursing homes respond to sexual abuse?

## 2. Materials and Methods

In order to answer our research question, we conducted a literature review. There are several forms of literature reviews. Grant and Booth [33] describe 14 different types. Critical review, overview, systematic review, and scoping review are some examples. Systematic literature review is the most known and most used of all review methods. A systematic literature review aims to systematically identify, critically appraise, and synthesize existing research on a specific area of knowledge. This involves exhaustive and comprehensive literature searching with explicit inclusion and exclusion criteria. The conduction of the review often follows specific guidelines and the quality of the selected articles is assessed. Scoping reviews also focus on completeness of literature searching on a specific area but does not require formal quality assessment [33]. In this study, we use some principles of a systematic review, such as exhaustive and comprehensive literature searching with explicit inclusion and exclusion criteria. But since we do not make a quality assessment of the selected articles, the study also has elements of a scoping review. Our study can thus be characterized as a mixture of a systematic review and a scoping review.

### 2.1. Inclusion and Exclusion Criteria

In the present review, the inclusion criteria were

- articles which exclusively address sexual abuse of older persons in nursing homes,
- articles based on either qualitative or quantitative methods,
- only original studies or reviews,
- written in English or any Scandinavian language,
- published in a scientific journal.

In the present review, the exclusion criteria were

- studies concerning sexual abuse of older persons living in their own homes, or studies concerning sexual abuse of both nursing home residents and older persons living in their own homes,
- studies on sexual abuse of nursing home residents less than 60 years, (We chose to include two articles where three out of 20 victims were less than 60 years [36] since the majority filled the inclusion criteria. One article [34] where 7 men were in their fifties was also included, since the further analyses did not distinguish on age.)
- studies concerning other types of abuse, like financial, physical, or psychological,
- studies concerning elder abuse in general.

### 2.2. Databases and Search Terms

Systematic searches in reference databases including Cinahl, Medline, OVID Nursing Database, ISI Web of Science, PsycINFO, Cochrane Library, and SveMed + were conducted from September 2012 until May 2013. The searches were based on the main search terms* seniors, aged, elderly, residence, nursing residence, nursing home, homes for the aged, institutions, long term care, sexual abuse, sex offences, *and* sexual assault.* Keywords were searched alone and combined in groups. Since we knew that there is little research on sexual abuse of older nursing home residents, the literature searching was not limited to year of publication. Through alert services from a selection of the databases: Cinahl, Medline, OVID Nursing Database, ISI Web of Science, and PsycINFO, we have since May 2013 received notifications when new records matched our search criteria. Until August 2014, no records have met our inclusion criteria.

### 2.3. Data Collection and Analysis

**Figure 1:**
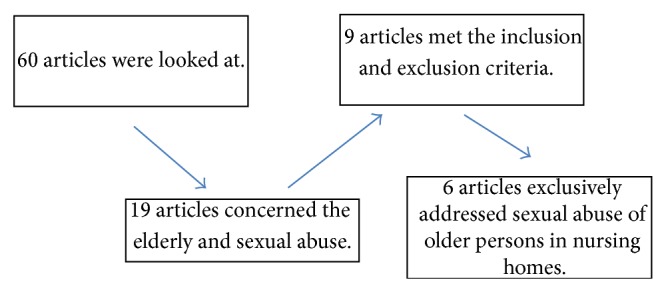
Phases of the selection of articles.

Based on the literature searches, 60 studies were looked at. Some of these turned out to be comments or letters and others were studies in which elderly and sexual abuse was mentioned in passing. 19 articles concerned the elderly and sexual abuse. 9 of these met the inclusion and exclusion criteria. A more thorough review showed that 3 articles also concerned sexually assaulting of elderly living at home in addition to sexual abuse in nursing homes; this did not appear in the abstracts. These 3 articles were then excluded and 6 articles were thus chosen for a deeper examination. All 6 articles were from the United States and published from 2000 to 2012. 3 were based on qualitative studies, 2 were quantitative, and 1 was a combination (see Figure 1).

## 3. Results and Discussion 

The articles are presented in Table 1. The table covers each study's purpose, method, participants, and main findings. Footnotes are used in the tables where there is a need for further explanation. The discussion section is related to the research questions presented in the introduction.

### 3.1. Definitions

Only two of the six articles included in the review present a clear definition on sexual abuse [21, 34]. In both articles the NCEA definition is used. All articles in the review are from USA and Ramsey-Klawsnik et al. [35] point to the fact that different states have their own definitions of sexual abuse. The problem caused by using different definitions is well documented by de Donder et al. [5], stating that this may influence the prevalence rates. This problem is also clearly demonstrated in a UK study by O'Keeffe et al. [8] when they write that: “The only reports of sexual abuse had to do with being talked to in a sexual way that had made the person feel uncomfortable and being touched in a sexual way against their will. These reports are at the less serious end of abuse and are more properly classified as harassment” (page 42). Others would, without hesitation, categorize this as sexual abuse. According to WHO [13] sexual abuse is defined as nonconsensual sexual contact of any kind with the older person. This is also in line with the NCEA definition. A universally accepted definition would be helpful in order to provide the world with more knowledge about sexual abuse of older residents in nursing homes.

### 3.2. Prevalence of Sexual Abuse against Older Residents in Institutions

None of the six selected articles can tell anything about the prevalence of sexual abuse of older residents in institutions in numbers. The data underlying the various studies are collected from cases of abuse reported to Adult Protective Services (APS) or other reporting agencies from various states in the United States. As far as we know, prevalence studies of sexual abuse in nursing homes have only been conducted as a part of larger studies, which include all types of abuse, for example, O'Keeffe et al. [8] and Kristensen and Lindell [6]. Since we also know that some studies categorize sexual abuse under the label physical abuse, for example, Eisikovits et al. [39], it is at this stage impossible to estimate the prevalence of sexual abuse of nursing home residents. It is obvious that more research is needed and that this specific type of elder abuse must be given more attention.

### 3.3. Types of Sexual Abuse

Four of the six articles describe the types of sexual abuse [21, 34, 36, 40]. The abuse reports showed that the older persons suffered from a variety of sexual abuse types, for example, anal and vaginal penetration and rape, oral/genital contact, and different kinds of verbal sexual abuse. The types of sexual abuse reported to be most common were sexualized kissing and fondling, as well as inappropriate sexual interest in the victim's body. The review also indicates that multiple types of abuse may occur in one single case and that the eldest women were more likely to experience multiple types of abuse [34].

### 3.4. Characteristics of Victims

The findings of the articles show that both male and female nursing home patients are victims of sexual abuse. However, in the majority of cases, violence against women was reported [21, 34, 35, 38]. WHO [13] states that official statistics vastly underrepresent male victims of sexual abuse, and this seems to be the case also for elder abuse. One article is focused only on male victims [34] and the authors emphasize the presence of older men as victims of sexual abuse. Could it be so that the older the age, the less important the gender issue, and factors like fragility, dependency, and cognitive decline become more important? More research on gender issues in elder abuse is needed.

The review shows that most victims of sexual abuse in nursing homes were cognitively impaired (dementia, Alzheimer's, stroke, and brain injury), had a psychiatric diagnosis and/or were physically frail (wheelchair, bedridden, paralyzed, and reduced mobility), and had somatic illnesses [21, 34, 35, 38]. The oldest patients (age 79–99 years) were more frequently subjected to sexual abuse [21, 34, 35, 38]. This could support the view that other factors than gender are more important in the oldest age group. This is also underlined in the WHO report [13], where it is, based on community-based prevalence studies, concluded that older men are at risk of abuse by spouses, adult children, and other relatives in about the same proportion as women.

### 3.5. Characteristics of the Perpetrator

Findings from the studies included in this review show that the alleged and confirmed perpetrators were predominantly men aged 18–80 +, but there were also a few women among the confirmed perpetrators [21, 34, 35, 38]. Nursing home staff and coresidents were the most common perpetrators [34–36]. The second most common perpetrator was a family member. Visitors known or unknown to the victim were less common [35]. In many cases the abuser had either cognitive impairment, psychiatric diagnosis, substance abuse, criminal history, or previous committed sexual abuse [21, 35, 36]. The study of Burgess et al. [36] found that all the offenders were driven by different motives. Two common denominators for all offenders were that they scored low on social competence and they looked for victims who were fragile and defenseless. Teaster et al. [34] question whether it is easier for investigators to confirm a resident as a perpetrator of sexual abuse rather than a facility staff member.

### 3.6. Consequences for Perpetrators and Victims

Findings from the studies [21, 34, 36] show that only a few perpetrators were held accountable and that the assault had no legal consequence, despite witnesses in several of the abuse situations. In cases where patients were abusers, the patients were transferred to other departments or other nursing homes [21, 35]. Where the perpetrators were a staff member they were either terminated from employment, placed on leave, or transferred to other jobs. Some got their name in the criminal records and a few were sentenced [21, 35, 36]. When it comes to victims of abuse, they either were moved to another department internally at the nursing home, were followed-up, or did not get any help at all. Very few received medical and psychological treatment after the assault. Many of the victims continued to be at risk of more abuse [21, 34, 35, 38].

Common consequences of sexual violence are mental health problems, including suicidal behavior [13]. Deaths associated with rape are known to occur, according to [13]. Data in the study made by Burgess et al. [38] shows that 11 out of 20 nursing home patients died within a year after the sexual assault. One cannot really say whether these deaths were a result of the assault or not, since many of the victims were old and frail. As regards the report of the abuse to the authorities, the most common reasons why the cases were not reported were lack of evidence and the victim's medical condition [21]. According to Ramsey-Klawsnik and Teaster [37] the APS employees who should investigate cases of abuse did not feel competent to perform the investigation. APS employees reported that they had little training in handling these types of cases. It is rare that there are witnesses to sexual abuse and the victims themselves are often in such a state cognitively and physically that investigating and collecting evidence is difficult. Most abuse cases were dismissed because of insufficient evidence, although APS workers said that the likelihood that a violation had occurred was present.

### 3.7. The Institutions' Response to Sexual Assault

According to the articles in the review [21, 34–38] most nursing homes did not handle situations of sexual abuse in an adequate way. Many nursing homes did not report further about sexual abuse or they delayed reporting to authorities. There was also a lack of documentation of abuse. Findings from Ramsey-Klawsnik and Teaster [37] showed, however, that some of the nursing homes responded adequately to the sexual abuse cases. The nursing homes managed to protect the victim from the perpetrator, reported promptly to the investigative bodies, and showed good cooperation. However, studies also showed that several nursing homes failed to provide medical assistance to patients who were victims of sexual abuse and to protect the victim from the perpetrator [37, 38]. Several of the staff minimized or ignored reporting sexual abuse. One of the articles in the review focuses mainly on prevention of abuse in health care facilities [37]. Their recommendations for facility response to alleged sexual abuse are amongst others immediate action (including immediate medical attention for victims), documenting detailed information, and collaborating with law enforcement. They also recommend training for staff regarding, for example, signs and symptoms, patterns of abuse, victim impact, perpetrator behaviors, and appropriate responses. WHO [13] states: “prevention starts with awareness” (page 142), and it emphasizes that health care providers, also in institutions, should receive basic training on the detection of elder abuse.

## 4. Conclusion

The purpose of this study was to assess the state of knowledge on the subject of sexual abuse against older nursing home residents through a literature review. Findings from the review show that sexual abuse occurs in nursing homes and that both older women and men are victims of this type of abuse. Perpetrators appear mainly to be staff and other residents and mainly to be men, but one needs to be aware of the fact that also women abuse both older men and older women. Nursing homes often show inadequate handling of abuse cases, and there is need for more knowledge among health professionals and guidelines for handling these types of cases. The fact that nursing home staff are not aware that this can happen, or have a hard time believing that it could have happened, can increase the nursing home resident's vulnerable position as potential victims of abuse. It makes it even more challenging to speak out about or to uncover sexual abuse.

Sexual abuse of older persons crosses the traditional gender, cultural, and role boundaries for victims and perpetrators [34]. There is a risk that sexual abuse against older persons is not taken seriously since they, due to ageism, may be seen as asexual. Thus, many see it as unlikely that sexual abuse may occur. This is according to Connolly et al. [41], expressed when health professionals avoid asking older people about sexual health and refuse to believe or respond to allegations of sexual abuse. Health care system, police, and the judicial system should, according to the authors, be trained on how to handle sexual abuse of the older persons in a way that respects and protects the victim and secures evidence and documentation.

Findings from the literature review show that there is a need for knowledge and further research on the topic of sexual abuse against older residents in nursing homes. Furthermore, there is a need for good policies and reporting systems, as an important step in seriously addressing sexual abuse against older persons. Further research should aim to reveal more about this taboo area.

## Figures and Tables

**Table 1:** Description of articles included in the review.

Article	Aim	Method	Participants	Main findings
Teaster and Roberto [21]	To investigate the characteristics and outcomes of sexual abuse against elderly women in nursing homes.	Data collection from APS case files^1^ of sexually abused elderly women in nursing homes collected over a four-year period.	50 female nursing home residents aged 70–89 years who have been victims of sexual abuse.	The most common form of sexual abuse was sexual kissing, fondling, and unwelcome sexual interest in the women's body. 90% of the perpetrators were male nursing home residents over 70 years. Three of the cases ended up in court and one perpetrator was convicted.

Teaster et al. [34]	To analyze the reported cases of sexual abuse against men in nursing homes and to understand the characteristics of sexual abuse, who victim and perpetrator are, and how cases are handled.	Data collection from APS case files and other regulatory agencies. Reporting from five states in the U.S., over a 6-month period.	24^2^ male nursing home residents from 50 to 93 years who have been sexually abused in nursing homes.	Over 80% of the victims had cognitive and physical deficits that limited their ability for self-care. Type of abuse: fondling 35%, unwanted sexual attention 27%, finger penetration of the anus 12%, and rape 9%. Of the suspected perpetrators were 75%^3^ staff and 25% other nursing home residents. Of 26% of the reported cases were 6 substantiated.

Ramsey-Klawsnik et al. [35]	To better understand the sexual abuse of the elderly in institutions. Furthermore, to examine the characteristics of the victim and the perpetrator and also the factors related with abuse situations.	Randomized selection of the reported cases of abuse by APS and other reporting agencies, from five U.S. states. Information from the MDS^5^ was used to examine the consequences for the victims over time.	96 female and 27 male nursing home residents aged 60–101 years, who had been sexually abused. 119 perpetrators.	The victims suffered from a number of cognitive and physical illnesses. Of the suspected perpetrators were most employees^4^ (*n* = 51) and other nursing home residents (*n* = 48). In 32 of the cases it was proved that the assault had taken place. No perpetrators were given legal punishment.

Burgess et al. [36]	To explore the characteristics of perpetrators.	Review of relevant documents retrieved from the courts. Of the 20 case files that were reviewed, the perpetrators were identified in 18.	18 perpetrators who had committed sexual abuse of nursing homes residents.	The perpetrators were from 16 to 83 years and either employees^6^ (*n* = 15) or nursing home residents (*n* = 3). Two common denominators for the perpetrators were that (1) they scored low on social competence; (2) they hunted victims who were frail and defenseless.

Ramsey-Klawsnik and Teaster [37]	To focus on sexual abuse of the elderly in institutions. Collect data about experiences and handling of specific cases and further recommendations to help the victims and prevent abuse.	Randomized selection of reported cases. Participants were interviewed by telephone.	46 employees working in the APS and other regulatory agencies in five states in the U.S., receiving reports of sexual abuse against elderly in institutions.	Participants had examined a total of 300 reported cases. Interviewees indicated that they had a lack of training in handling these types of cases and called for guidance, knowledge, and education in the area.

Burgess et al. [38]	Drawing attention to sexual abuse of the elderly and to focus on victim impact.	Review of relevant documents retrieved from the court system, including records from nursing homes, interview, and video interview with victims.	18 female and 2 male nursing home residents subjected to sexual abuse.	11 of the victims died within 12 months after the assault. Most of the victims were cognitively or neurologically impaired. Half of the victims were examined medically. The victims suffered from anxiety, fear, and withdrawal and refused help with personal hygiene.

^1^Adult Protective Services (APS) is a state agency and its function is to assist the elderly who are prone to or have been victims of various types of abuse. They start preliminary investigation and assist the elderly in abuse cases.

^2^Started with 26, two fell out, no explanation.

^3^One woman was confirmed as the perpetrator.

^4^Three women were confirmed as the perpetrator

^5^Minimum Data Set, a public reporting system for cases of suspected sexual abuse. Medical database.

^6^One woman was confirmed as the perpetrator.
